# Schonende operative Technik bei posttraumatisch eingewachsenen Fingernägeln

**DOI:** 10.1111/ddg.15698_g

**Published:** 2025-12-11

**Authors:** Simona Sabulyte, Galina Balakirski, Christoph R. Löser

**Affiliations:** ^1^ Zentrum für Dermatologie Allergologie und Dermatochirurgie Universitätsklinikum Wuppertal Universität Witten/Herdecke, Wuppertal; ^2^ Hautklinik Hauttumorzentrum Klinikum der Stadt Ludwigshafen gGmbH Ludwigshafen am Rhein

**Keywords:** eingewachsener Nagel, Nagelchirurgie, Nagelverletzung, posttraumatische Nagelveränderungen, Unguis incarnatus, ingrown nail, nail trauma, nail surgery, post‐traumatic nail changes, Unguis incarnatus

## EINLEITUNG

Der Fingernagel hat eine große ästhetische Bedeutung und ist auch funktionell gefährdet durch grobe chirurgische Manipulation. Bei Anwendung traumatischer Operationsmethoden resultieren regelhaft irreversible Schäden mit funktioneller und/oder ästhetischer Beeinträchtigung.^1^

Im Vergleich zu eingewachsenen Zehennägeln ist das Krankheitsbild auch in der Literatur kaum präsent. Deshalb zeigen wir in zwei Fallbeispielen schonende Techniken zur operativen Behandlung bei posttraumatisch eingewachsenen Fingernägeln.

## TECHNIK

### Fallbericht 1

Ein 44‐jähriger Handwerker berichtete über Schmerzen am linken Mittelfinger, die ihn sowohl im Berufsleben als auch im Alltag beeinträchtigen. Bei einem Enchondrom der distalen Phalanx, das nach mehrfachen Spontanfrakturen festgestellt wurde, erfolgte vor etwa 8 Jahren eine Enchondromausräumung und Auffüllung des Knochendefektes mittels autogener Spongiosatransplantation vom Radius. Nach einem Rezidiv‐Enchondrom wurde vor 2 Jahren der Eingriff wiederholt und der Zugang zum Knochen durch das Nagelbett gewählt. Dabei kam es initial zum Verlust der Nagelplatte. Bereits wenige Monate nach dem Eingriff kam es zu zunehmenden Beschwerden am nachwachsenden Nagel.

**ABBILDUNG 1:**
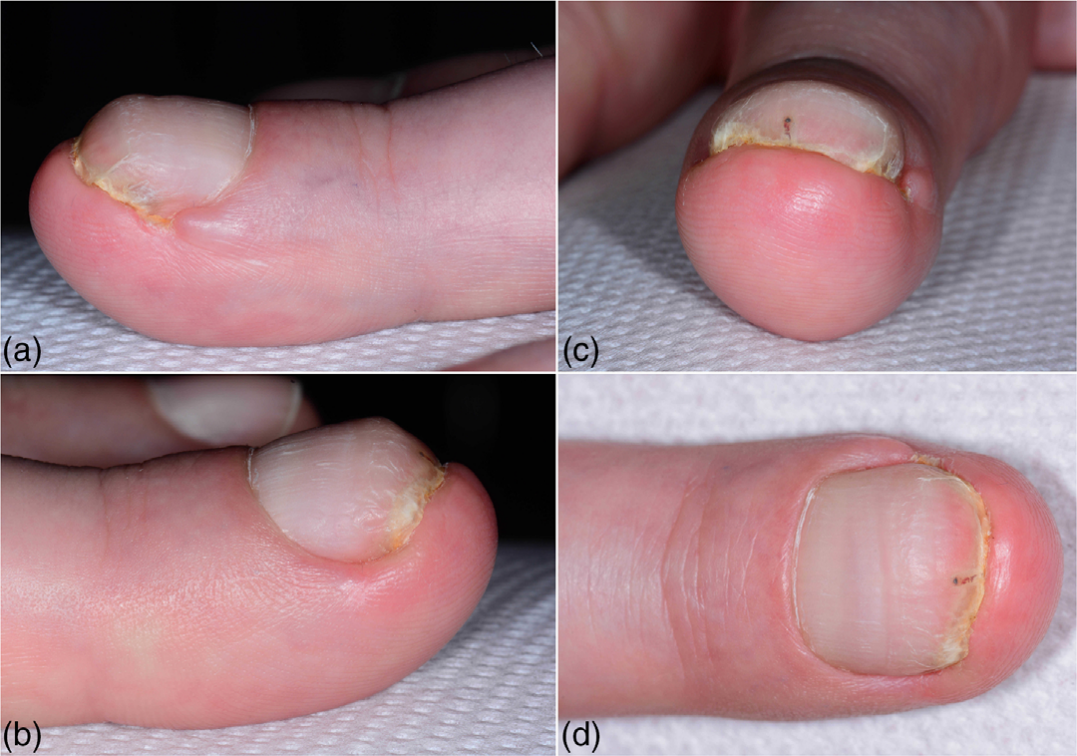
Präoperativer Befund mit Zangennagel und distalem Einwachsen des Nagels am linken Mittelfinger. (a, b). Die seitliche Ansicht zeigt deutliches laterales Einwachsen der Nagelplatte. (c, d) Die Ansicht von vorne und oben zeigt das distale Einwachsen des Nagelbetts in die Fingerkuppe durch die auch in Längsrichtung gekrümmte Nagelplatte.

**ABBILDUNG 2:**
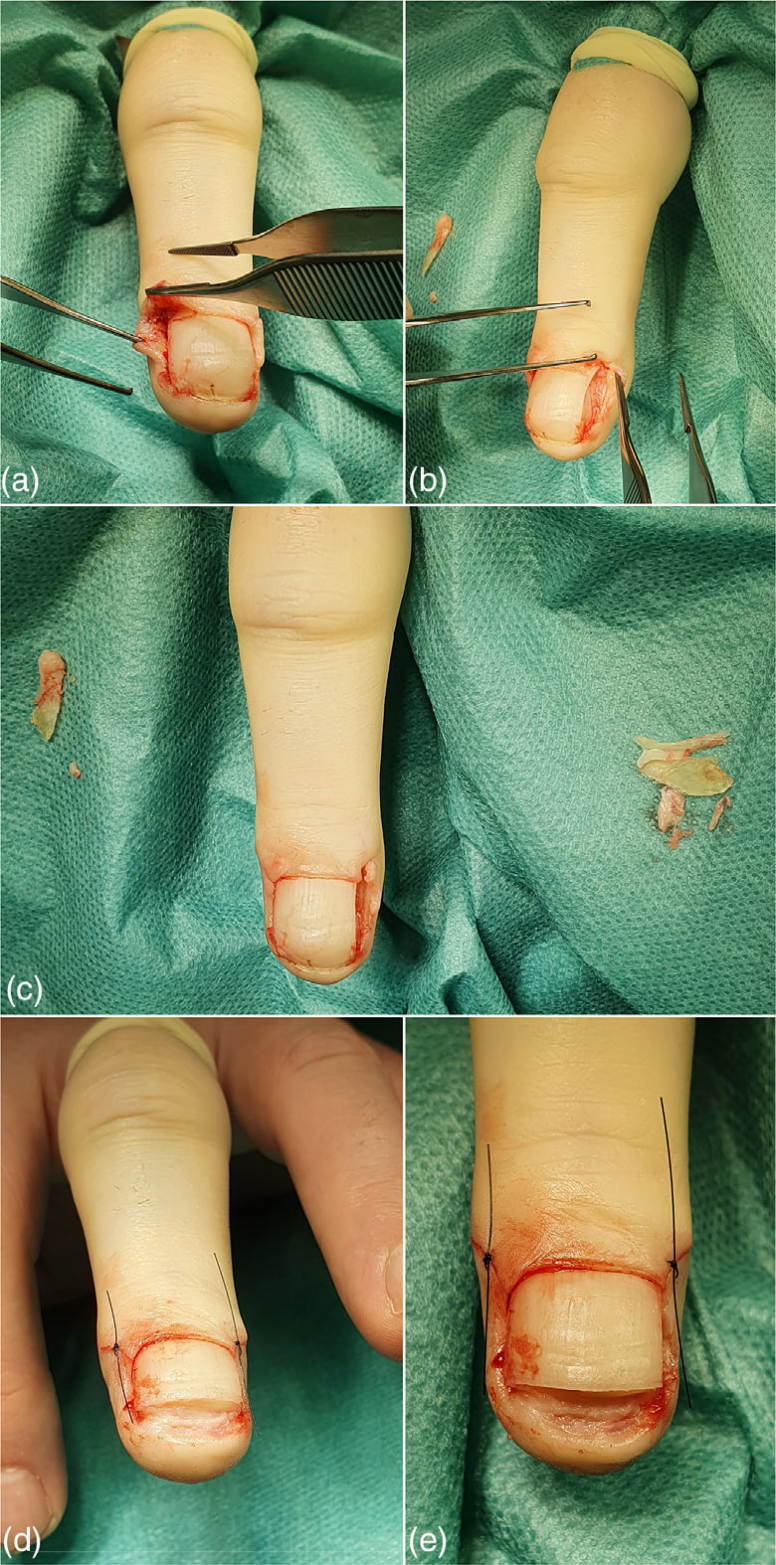
(a–c) Intraoperative Darstellung der Teilresektion der lateralen Nagelplatten und der seitlichen Matrixhörner sowie (d, e) distale Resektion der Nagelplatte und oberflächlicher Nagelbettanteile. Zu beachten ist, dass Matrixhornresektate hier nicht dargestellt wurden.

Klinisch zeigte sich ein deutlicher Zangennagel mit seitlichem sowie distalem Einwachsen (Abbildung 1a–d). In Leitungsanästhesie nach Oberst erfolgte eine Teilresektion der lateral eingewachsenen Nagelplatte und Entfernung der seitlichen Matrixhörner (Abbildung 2a–c) sowie eine distale Resektion der nach unten gekrümmten Nagelplatte mit oberflächlichen Anteilen des Nagelbettes (Abbildung 2d–e).

**ABBILDUNG 3:**
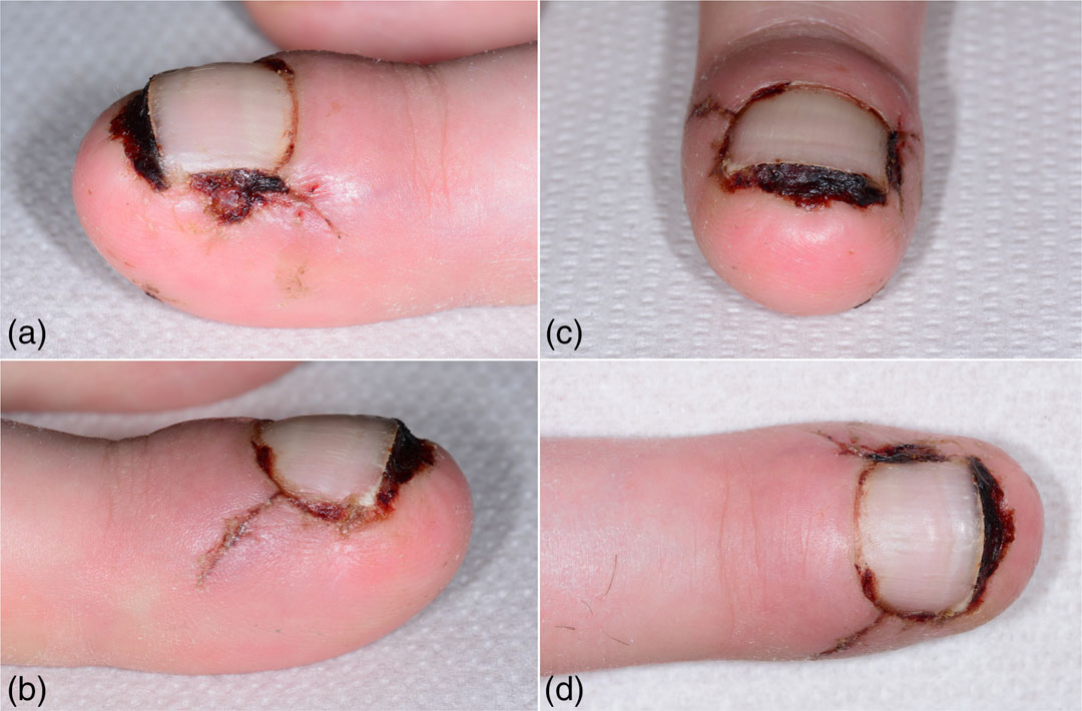
Klinische Verlauf beim Fadenzug etwa 2 Wochen postoperativ.

**ABBILDUNG 4:**
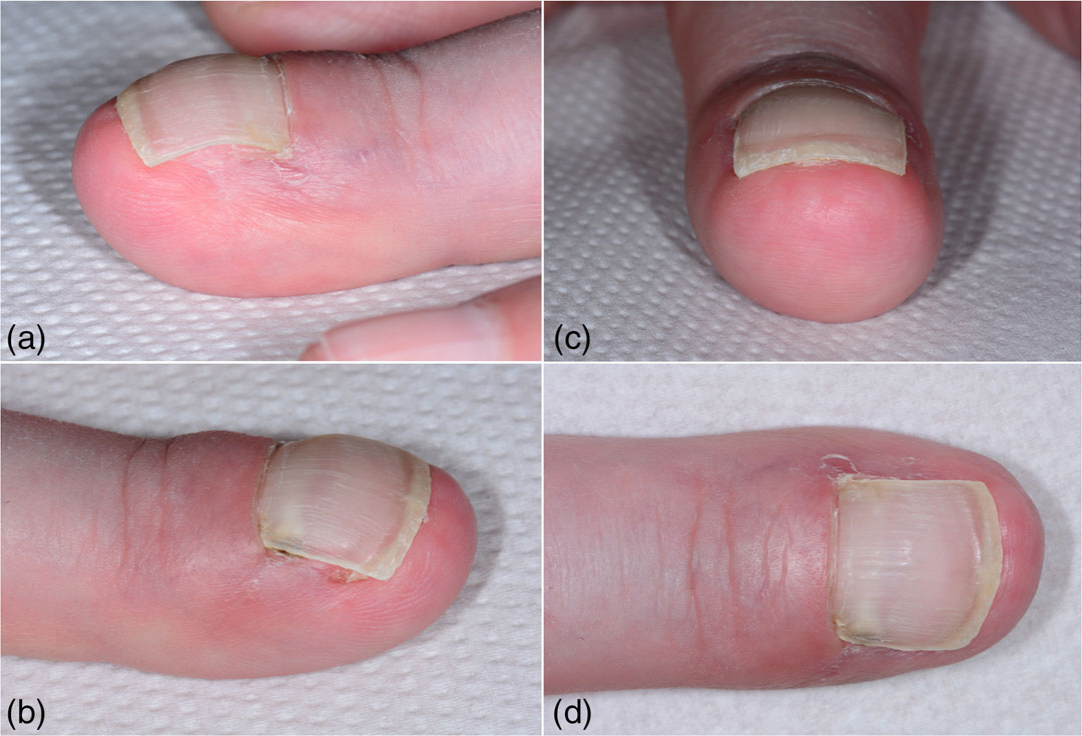
Klinischer Befund 8 Wochen postoperativ. Der Nagel bedeckt vollständig das Nagelbett ohne Hinweis auf das erneute Einwachsen in den distalen Nagelwall.

Beim Fadenzug etwa 2 Wochen postoperativ (Abbildung 3a–d) sowie bei der Verlaufskontrolle 8 Wochen nach dem Eingriff zeigte sich ein regelrechter Verlauf (Abbildung 4a–d). Nach Abheilung war der Patient erstmals wieder völlig schmerzfrei.

### Fallbericht 2

**ABBILDUNG 5:**
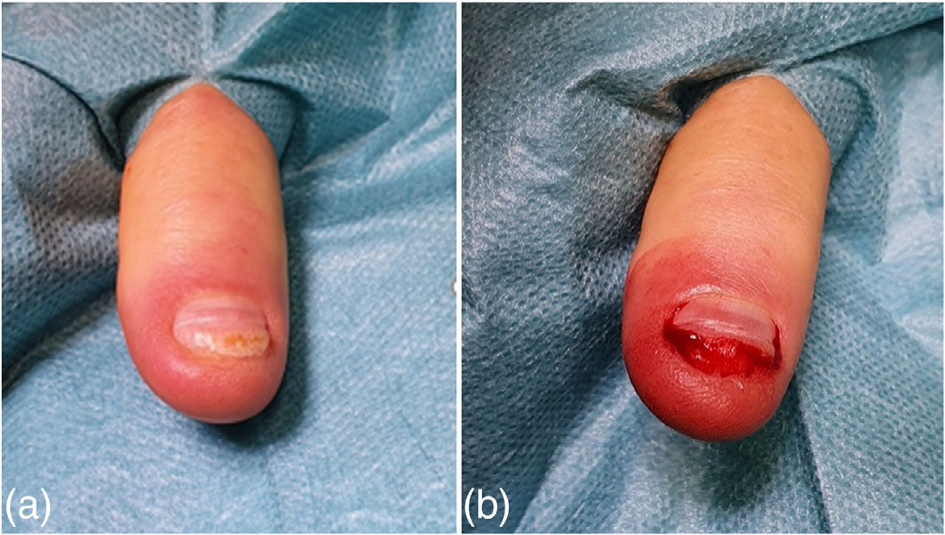
(a) Präoperativer Befund mit einem schmerzhaften, distalen Einwachsen der Nagelplatte bei beginnender Retronychie sowie (b) operative Korrektur mittels Kürzung der Nagelplatte mit Entfernung des eingewachsenen Nagelabschnittes sowie Anfrischen des Nagelbettes.

**ABBILDUNG 6:**
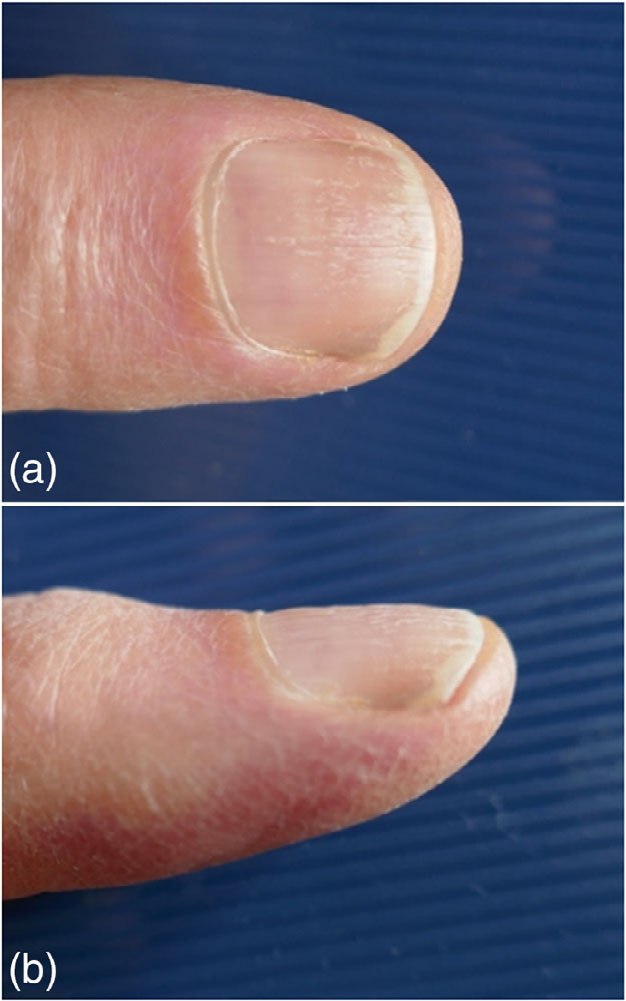
(a, b) Verlaufskontrolle mit vollständigem Nachwachsen der Nagelplatte über das Nagelbett wenige Monate postoperativ (Aufnahmen des Patienten, mit freundlicher Genehmigung).

Bei einem 74‐jährigen Patienten erfolgte nach einer Quetschverletzung durch eine Heckklappe eine Nageltrepanation, was im Verlust der Nagelplatte am rechten Daumen resultierte. Beim Nachwachsen der Nagelplatte entwickelte sich mittig ein sehr schmerzhaftes, distales Einwachsen der Nagelplatte mit beginnender Retronychie (Abbildung 5a). In Leitungsanästhesie nach Oberst erfolgte die Entfernung des eingewachsenen Nagelabschnittes und Anfrischen des Nagelbettes (Abbildung 5b). Dem Patienten wurde eine Nachbehandlung mittels rehydrierender Pflege des Nagelbettes und Massagen in Richtung des Nagelwachstums erklärt. Bei der Verlaufskontrolle einige Monate nach dem Eingriff zeigte sich ein regelrechter Verlauf mit komplettem Nachwachsen des Nagels und völliger Schmerzfreiheit (Abbildung 6a,b).

## DISKUSSION

Eingewachsene Zehennägel stellen ein häufiges und unter Fachärzten für Dermatologie bekanntes Krankheitsbild dar.^2^, ^3^ Der eingewachsene Fingernagel ist dagegen eine relativ seltene, weniger bekannte und kaum in der Literatur beschriebene Entität.^4^ Das Einwachsen der Fingernägel wird insbesondere bei der chronischen Paronychie unter Therapie mit *Epidermal‐Growth‐Factor‐Receptor* (EGFR)‐Inhibitoren beobachtet oder posttraumatisch.^4^

Für die eingewachsene Zehennägel wurde eine Unterteilung in Subtypen vorgeschlagen,^5^ die grundsätzlich auch für die Fingernägel benutzt werden kann, da sie lediglich der Beschreibung der Problemlokalisation dient: Distales Einwachsen (*distal embedding*), subkutan eingewachsener Nagel bei zu breit angelegter Nagelplatte, Hypertrophie des seitlichen Nagelfalzes und Zangennagel.^5^ Insbesondere nach einem vorangegangenen Trauma mit partiellem oder vollständigem Nagelverlust kann es zu einer übermäßigen Krümmung der nachwachsenden Nagelplatte im Längsverlauf kommen und somit zum schmerzhaften Einwachsen des freien Randes der Nagelplatte in den distalen Nagelwall.^6^ In solchen Fällen kann, wie in beiden Fallbeispielen dargestellt, die Teilresektion der Nagelplatte und der oberflächlichen Anteile des Nagelbettes zur Verbesserung des Zustandes führen. Die durch das distale Einwachsen der Nagelplatte resultierende längsgerichtete Hyperkurvatur des Nagelbettes kann dabei dem Ursprungszustand, parallel zur distalen Phalanx, angenähert werden. In den meisten Fällen wird durch weiteres Nagelwachstum die ursprüngliche Nagelform gleichmäßig über das Nagelbett hinweg wiederhergestellt.^7^ Eine wichtige Empfehlung nach der Korrektur des distal eingewachsenen Nagels ist, freiliegende Nagelbettanteile mit geeigneten Externa (beispielsweise Vaseline) täglich vor Austrocknung zu schützen und die Fingerkuppe gegebenenfalls vom Finger weg zu massieren, um einer Retraktion und damit einer erneuten Verkürzung des Nagelbettes entgegen zu wirken.^8^, ^9^ Konservative Therapieeinsätze mit Massieren und Tapen des distalen Nagelwalls sind ebenfalls beschrieben, ein Erfolg ist allerdings in der Regel erst nach mehreren Monaten sichtbar^8^, zudem ist die Dauer der Remission nach Beendigung der konservativen Maßnahmen unklar. Die Autoren empfehlen daher die beiden Vorgehensweisen, atraumatische Operationsmethode und konservative Nachbehandlung zu kombinieren, um eine nachhaltige Besserung der Symptomatik zu erreichen.

Zusammenfassend gibt es auch bei chronisch schmerzhaftem Einwachsen der Nagelplatte am Fingernagel minimal‐invasive Korrekturoptionen, die, wie in den Fallbeispielen demonstriert, aufwändige Korrekturen des Nagelbettes (beispielsweise Froschmaulplastik) oder heute obsolete Verfahren (wie ungezielte Nagelextraktion) zu vermeiden helfen.

## DANKSAGUNG

Open access Veröffentlichung ermöglicht und organisiert durch Projekt DEAL.

## FINANZIERUNG

Dr. med. Simona Sabulyte erhielt das Hospitationsstipendum zur Förderung der Dermatochirurgie der Deutschen Dermatologischen Gesellschaft (DDG) und der Deutschen Gesellschaft für Dermatochirurgie (DGDC) für das Jahr 2024.

## INTERESSENKONFLIKT

Keiner.
